# Predicting neurological outcome after cardiac arrest by combining computational parameters extracted from standard and deviant responses from auditory evoked potentials

**DOI:** 10.3389/fnins.2023.988394

**Published:** 2023-02-15

**Authors:** Aymeric Floyrac, Adrien Doumergue, Stéphane Legriel, Nicolas Deye, Bruno Megarbane, Alexandra Richard, Elodie Meppiel, Sana Masmoudi, Pierre Lozeron, Eric Vicaut, Nathalie Kubis, David Holcman

**Affiliations:** ^1^Applied Mathematics and Computational Biology, Ecole Normale Supérieure-PSL, Paris, France; ^2^Medical-Surgical Intensive Care Department, Centre Hospitalier de Versailles, Le Chesnay, France; ^3^CESP, PsyDev Team, INSERM, UVSQ, University of Paris-Saclay, Villejuif, France; ^4^Department of Medical and Toxicological Critical Care, APHP, Lariboisière Hospital, Paris, France; ^5^INSERM U942, Paris, France; ^6^INSERM UMRS 1144, Université Paris Cité, Paris, France; ^7^Service de Physiologie Clinique-Explorations Fonctionnelles, APHP, Hôpital Lariboisière, Paris, France; ^8^LVTS UMRS 1148, Hemostasis, Thrombo-Inflammation and Neuro-Vascular Repair, CHU Xavier Bichat Secteur Claude Bernard, Université Paris Cité, Paris, France; ^9^Unité de Recherche Clinique Saint-Louis- Lariboisière, APHP, Hôpital Saint Louis, Paris, France

**Keywords:** coma, electroencephalography, automatic classification algorithm, machine learning, neurological prognosis

## Abstract

**Background:**

Despite multimodal assessment (clinical examination, biology, brain MRI, electroencephalography, somatosensory evoked potentials, mismatch negativity at auditory evoked potentials), coma prognostic evaluation remains challenging.

**Methods:**

We present here a method to predict the return to consciousness and good neurological outcome based on classification of auditory evoked potentials obtained during an oddball paradigm. Data from event-related potentials (ERPs) were recorded noninvasively using four surface electroencephalography (EEG) electrodes in a cohort of 29 post-cardiac arrest comatose patients (between day 3 and day 6 following admission). We extracted retrospectively several EEG features (standard deviation and similarity for standard auditory stimulations and number of extrema and oscillations for deviant auditory stimulations) from the time responses in a window of few hundreds of milliseconds. The responses to the standard and the deviant auditory stimulations were thus considered independently. By combining these features, based on machine learning, we built a two-dimensional map to evaluate possible group clustering.

**Results:**

Analysis in two-dimensions of the present data revealed two separated clusters of patients with good versus bad neurological outcome. When favoring the highest specificity of our mathematical algorithms (0.91), we found a sensitivity of 0.83 and an accuracy of 0.90, maintained when calculation was performed using data from only one central electrode. Using Gaussian, K-neighborhood and SVM classifiers, we could predict the neurological outcome of post-anoxic comatose patients, the validity of the method being tested by a cross-validation procedure. Moreover, the same results were obtained with one single electrode (Cz).

**Conclusion:**

statistics of standard and deviant responses considered separately provide complementary and confirmatory predictions of the outcome of anoxic comatose patients, better assessed when combining these features on a two-dimensional statistical map. The benefit of this method compared to classical EEG and ERP predictors should be tested in a large prospective cohort. If validated, this method could provide an alternative tool to intensivists, to better evaluate neurological outcome and improve patient management, without neurophysiologist assistance.

## Introduction

Sudden death by cardiac arrest (CA) is a major public health issue, affecting 55 patients out of 100,000 with nearly 40,000 cases per year in France (Sfar, 2007). Five to 30% of the patients resuscitated after CA are alive at 1 year (Pell, 2003; Carr et al., 2009; Chin et al., 2022). Despite the use of veno-arterial extracorporeal cardiopulmonary resuscitation (VA-ECPR), a contemporary resuscitation approach that increases patients’ survival, prognosis remains grim (Miraglia et al., 2020). Favorable outcome after discharge relies mainly on the prognostic value of brain injury that outweighs the combined effects of all other terminal organ failures (Roberts et al., 2013; Rossetti et al., 2016).

Assessment of neurological damage is usually performed 48–72 h after CA and, optimally, after interruption of sedative drugs (Nolan et al., 2021). The evaluation is multimodal and combines, according to available local resources, clinical evaluation (Glasgow Coma Scale, photomotor and pupillary reflexes), biological markers of neural cell necrosis (NSE and S100bêta proteins), cerebral Magnetic Resonance Imaging and electrophysiological studies including electroencephalography (EEG), somatosensory evoked potentials (SSEP) and auditory evoked potentials (AEP). EEG analysis allows grading of post-anoxic encephalopathy (Synek, 1988), “highly malignant” EEG pattern (Westhall et al., 2016; André-Obadia et al., 2018), being associated with the least favorable prognosis. The absence of EEG reactivity can predict mortality and poor outcome. However, it is prone to large inter-rater variability when only determined using visual analysis. For this reason, quantitative methods developed to objectively measure EEG reactivity are promising (Duez et al., 2018; Admiraal et al., 2020; Bouchereau et al., 2022) and somatosensory and auditory evoked potentials can also be used to improve the accuracy of the patient outcome. The absence of cortical N20 response at SSEP after stimulation of median nerves has an almost 100% specificity for non-awakening prediction (Sandroni et al., 2014), while the presence of a “mismatch negativity” (MMN), an endogenous long latency negative potential at AEP (Rohaut et al., 2009) would rather indicate a good prognosis. The absence of cortical N20 response at SSEP after stimulation of median nerves has an almost 100% specificity for non-awakening prediction (Sandroni et al., 2014). The presence of a “mismatch negativity” (MMN), an endogenous long latency negative potential at AEP (Rohaut et al., 2009) would rather indicate a good prognosis.

Mismatch negativity consists in recording cortical potentials in response to auditory stimulation delivered by earphones, using electrodes placed on the scalp. The MMN (or N200), is a negative event-related potential (ERP) that occurs between 100 and 250 ms predominantly over the frontocentral scalp area and is obtained by the subtraction of oddball auditory stimuli (called deviant stimuli) randomly intermixed with repetitive frequent auditory stimuli also called standard or non-deviant stimuli. Thus, MMN reflects the ability to detect automatic auditory violations, but sensitivity to predict awakening is low (56%) with a high 93% specificity (Naccache et al., 2005). Because of lack of sensitivity in the ICU when interpreted only by visual analysis (present or absent) (Azabou et al., 2018), complementary statistical methods have been developed to analyze MMN more accurately, increasing thus the positive predictive value for awakening (Pfeiffer et al., 2017), at the cost of extension of the time of interpretation. Thus, multimodal approaches combining several prognostic factors of post-anoxic coma have been proposed (Bassetti et al., 1996; Fischer et al., 2006; Kim et al., 2012; Oddo and Rossetti, 2014) but the choice of these approaches has not yet succeeded to lead to automatic and predictive analyses.

Taking advantage of the considerable amount of information obtained at AEP, we conducted an explorative study in which we applied a machine learning classification approach based on EEG features arising from the distribution of the ERP fluctuations responses during the 20 min-recording, rather than to interpret the MMN as a binary response. We used data already acquired from a homogeneous cohort of patients admitted in the intensive care unit after CA and who all had EEG, SSEP and AEP recordings within 6 days after admission. We identified specific features from AEP, considering responses to standard and deviant auditory stimulations independently. Using a step-by-step data processing, we finally reported combined features in two-dimensional map where we observed that patients were clustered into two groups corresponding to a different outcome at discharge whether they were able to follow verbal command or not. We then estimated the probability for a patient to be classified into one of the two groups at the acute phase using several classifiers.

## Patients and methods

### Procedure

This study is a retrospective single-center study performed in 29 consecutive patients between January 2014 and March 2016, successfully resuscitated after CA, with persistent coma between the 3rd day and 6th day following admission in the Department of Medical and Toxicological Critical Care in Lariboisière Hospital (Paris), and who completed EEG, SSEP, and AEP recordings. From AEP recordings, we extracted individual features, and using a novel analysis method, we aimed to classify patients into two categories: communicating patients (assumed to have a good neurological outcome) and deceased or non communicating patients, according to their capacity to follow verbal command at discharge.

This study is an ancillary study of the PHRC CAPACITY AOR10109 and was approved by the ethics committee (Comité de Protection des Personnes, CPP Paris IV #2012/22). As this AEP processing was performed secondarily, physicians who were in charge of the patients could not have access to these data. Withdrawal of life-sustaining therapies was performed according to the usual guidelines (Société de réanimation de langue française., 2010).

### Clinical data

Cardiac arrest characteristics, in-hospital management and outcome data were collected according to Utstein method by the intensivists in charge during hospitalization (Perkins et al., 2015). During ICU stay, the following data were collected: age, sex, past medical history; presumed etiology categorized into non-cardiac, cardiac and undetermined; shockable rhythm; time from collapse (CA) to return of spontaneous circulation (ROSC) dichotomized into ≤25 or >25 min (Oddo et al., 2008); interval from the time of collapse (presumed time of CA) to basic and/or advanced life support, defined as no-flow duration, and the interval from the beginning of life support until the return of spontaneous circulation or termination of resuscitative efforts, termed low-flow duration; hypothermia; Glasgow Coma Scale (GCS) on admission; SAPS II (Simplified Acute Physiology Score) (Le Gall et al., 2005); sedation.

Good neurological prognosis was defined by appropriate response to verbal command. Moreover, the Glasgow Outcome Scale Extended (GOS-E) was retrospectively collected at 3–6 months, when information was available.

Because of the retrospective design of our study, withdrawal of life-sustaining therapies decisions had been taken before our new analysis. They were multimodal and based upon European guidelines ERC-ESICM (2014).

### Electrophysiological data

We used electrophysiological data acquired between day 3 and day 6 following admission, in order not to include patients with early predictable death. However, most of them had previous EEG recording in the first 48 h. All data were analyzed or double-checked by specialists in clinical neurophysiology with at least 10 years’ experience.

#### EEG

Digital electroencephalography (EEG) recordings were performed for at least 20 min, with 21 scalp electrodes positioned according to the standard 10–20 system placement, reformatted to both bipolar and off-head referential montages, with filter settings at 0.3 and 70 Hz. Repetitive bilateral auditory and painful stimulations were systematically performed. These stimulations aimed to evaluate EEG reactivity and performed according to a standardized protocol for auditory (clapping noise, patient’s name and patient’s surname) and nociceptive stimulations (nail bed pressure plied to each upper limb) regularly applied in the same order. EEG was classified according Synek’s classification (Synek, 1988), which defines precisely the five major EEG patterns based on the allocation of patients into five principal categories regarding their significance for survival (optimal, benign if persistent, uncertain, malignant if persistent and fatal).

#### Somatosensory evoked potentials

Median nerves were stimulated at the wrist to an intensity of 4–5 mA, greater than that needed to evoke a muscular response, and in the case of the use of neuromuscular blocking, the ERB potential amplitude was used to estimate the intensity of the stimulation. Pulse duration was 0.2 ms and stimulus rate 3 Hz. Active electrodes were placed at Erb’s point and C3 and C4 points. At least two repetitions (averages of 300 responses) were performed to assess the reproducibility of the waveforms. N20 cortical response was dichotomized into absent or present.

#### Mismatch negativity

The auditory event related potentials were elicited using the classical odd-ball paradigm technique as already described (Fischer et al., 1999).

Event-related potentials were recorded with active electrodes (in an electrode cap) positioned at Fz, Cz, C3, C4 according to the International 10–20 system, reference electrode at the mastoid and ground reference at the forehead. Acoustic stimuli were delivered through earphones binaurally using a randomly intertwined sequence of standard and deviant stimuli in the proportion of 86 and 14%, respectively. Standard stimuli were delivered at a frequency of 800 Hz and lasting 75 ms each. Deviant stimuli were delivered at a frequency of 880 Hz and lasting 30 ms each to distinguish them from the standard stimuli (Fischer et al., 1999; Chausson et al., 2008; Comanducci et al., 2020). The interstimulus interval was 500 ms. EEG signals were band-pass filtered (0.5–75 Hz) using a time window of 500 ms. Each recording was performed during 20 min. Presence/absence of MMN defined as the negative peak obtained between the difference between deviant and standard response occurring in the 100–300 ms time interval following stimulation. In our experience, MMN is delayed in those critically ill and sedated patients, which explains this relatively wide time window.

#### Electrophysiological analysis

All data were analyzed by at least two different neurophysiologists, blind to the neurological outcome of the patients. When artifacts were too numerous leading to unreliable conclusion, data were not considered.

### Statistical analyses for demographic and clinical data

In each group (good or bad neurological outcome), results of clinical and neurophysiological examinations were expressed as mean ± SD [min-max] and median [IQR 25–75], when appropriate. Statistical analyses were performed with Prism 5 software (Prism 5.03, GraphPad, San Diego, USA). Comparison of frequencies in each group was analyzed by the Fisher’s exact test. A value of *p* < 0.05 was considered statistically significant.

### Signal processing, features identification, and classification

This section is divided in three parts: 1-Signal processing, 2-Feature identification and 3-Classification using a two-dimensional map. Without *a priori* consideration, we considered specific features in a 1 s duration window, then in a shorter window of 500 ms, then at last in 320 ms, which contains the relevant features and gave similar results compared to the two other time windows. This time interval was chosen large to start (in order to take in consideration the maximum amount of information, then was restricted to the smallest time interval that still contained the whole information. The data corresponding to the responses obtained from standard and deviant auditory stimulations were considered independently, regardless of the MMN that was not considered here, and mathematical processing was applied as for any signal, independently of its potential significance. We chose specific independent features for the standard and deviant stimulations that allowed increasing the robustness of the results, and preventing a potential bias by choosing a single set of parameters. At last, we combined them into a two-dimensional map, and patients formed two clusters according to their outcome. All these steps were determined without *a priori* knowledge of the patient’s prognosis.

#### Signal processing

**FIGURE 1:**
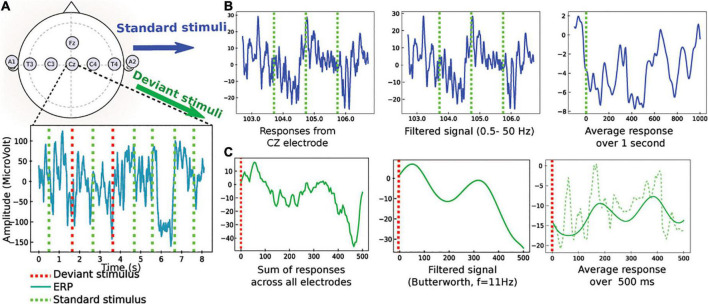
Pre-processing of the evoked auditory responses to standard and deviant stimuli (an example of data obtained from the CZ electrode is given for standard stimuli and an example from all electrodes is given for deviant stimuli). **(A)** Upper: standard position of the EEG electrodes. Lower: EEG traces during a protocol mixing standard (green) and deviant (red) stimulations. **(B)** Sample of standard stimuli (blue) the EEG signal from CZ-electrode is filtered 0.5–50 Hz. The output is an average filtered response over 1 s. **(C)** Pre-processing of deviant stimuli: (1) the signal is summed over electrodes, (2) a low-pass filter is applied (butterworth with *n* = 2, cutoff frequency at 10 Hz), (3) average filtered response (continuous green) in a window of 500 *ms* to a deviant stimulus, computed after synchronization to the stimulus. The non-filtered average response is also shown (dashed line).

Auditory evoked potential obtained with standard and deviant auditory stimulations were exported in the European Data Format (EDF), which is a simple and flexible format for storage of multichannel biological and physical signals, then anonymized through a specific software we designed. Analyses were performed on all four active electrodes then on one single Cz (central) electrode in order to see if we could obtain similar results with a simplified electrodes setting. *To quantify the auditory evoked responses recorded from post* CA patients in the intensive care unit, we studied separately standard and deviant responses (Figure 1), which is a novel and different paradigm compared to the classical MMN. We took into account the total 20 min extracted data, instead of the short interval response occurring in [100–300] ms following auditory stimulation. We filtered the signal in the [0.5–50] Hz band. Finally, all standard and deviant stimulations were averaged leading to a response in the time interval [0−1000]*ms*,[0−−500ms], and [0–320 ms], without difference in the analysis of the time intervals. To note, there was no difference either in the responses when they were computed in the interval [20–320 ms] that still contained the relevant information. Therefore, we converged to compute all statistics over a time window of [20–320] for all sounds, and results are presented in this interval.

We first focused on the ERP responses to standard periodic auditory stimuli, every 1s. We filtered the time series *X*(*t*) using a Butterworth bandpass filter (*n* = 4) in the frequency range 0.5–50 Hz and obtained the output *X*_*f*_(*t*). Finally, we averaged the signal in the time interval[0−1]*s*, ensuring that auditory stimuli were produced at time *t* = *nT* (*T* = 1s) leading to the response


(1) $$X_{p}\,\left(s\right)=\frac{1}{N}\,\sum_{1}^{N}X_{f}\,\left(s+n\,T\right),s\in \left[0-1\right]$$

where *N* is the number of periods (typically of the order 10^3^). This preliminary procedure therefore allowed obtaining an average response *X_p_* that highlights any possible deterministic feature present in the response. We applied a similar averaging procedure for deviant stimuli (see below and Figure 1).

##### Analysis of responses to standard stimuli

For the analysis of standard stimulation, we divided the 20 min recording into two parts (two consecutive sequences of 10 min), to explore a possible adaptation between the first part of the acquisition and the last part. If patients’ responses to auditory stimulations are able to fluctuate, this could indicate a better prognosis. This “reactivity” or ability to adapt is already used when interpreting the EEG in the ICU and indicates a better neurological outcome. We have introduced two parameters to that possible adaptation analysis: the variance of the signal computed over 10 min and the correlation between the two parts of the signal. The main parameters we extracted to study the response to standard stimulations were defined as follows:

We computed the standard deviation σ_*X*_ of the signal in the time interval [20−320]*ms*.


(2) $$\sigma_{X}^{2}=\frac{1}{t_{2}-t_{1}}\,\int_{t_{1}}^{t_{2}}{\left(X\,\left(t\right)-<X\,\left(t\right)>\right)}^{2}\,dt,$$

where *t*_2_ = 320 *ms* and *t*_1_ = 20 *ms*, and *X*(*t*) is the average of the X variable over the time [t1, t2]. This time interval corresponds to time scales of the neural networks involved in cognitive tasks.

We then divided the acquisition time of 20 min into n equal parts. For *n* = 2, we got [1−10]*min* and [10−20]*min*. We averaged the signals on each of these periods to obtain two responses *X*(*t*) and *Y*(*t*) in the interval [0−1]*s*. We computed the time correlation or similarity in [20, 320]*ms* of these two signals:


(3) $$r\,\left(X,Y\right)=\frac{<\left(X\,\left(t\right)-<X>\right)\,\left(Y\,\left(t\right)-<Y>\right)>}{\sigma_{X}\,\sigma_{Y}},$$

where < . > represents this time average.

We therefore used these two parameters to define the space state for the coordinates a patient: (1) the standard deviation computed over the entire sample of 20 min and (2) the similarity, computed in Eq. 3. These coordinates define a mathematical state space, which is not a specific of the medical state of the patient.

##### Analysis of responses to deviant stimuli

Deviant stimuli are random stimuli that account for 14% of the entire responses. The approach used for standard responses analysis is not well suited for deviant stimulations, as we did not expect any adaptation in time of such a random motif. We choose two parameters that are classically used for analysing oscillatory signals, the number of extrema and the total variation for the oscillation. We filtered the resulting signal *X_d_* using a lowpass Butterworth filter (*n* = 2) with a cut off frequency at 10 Hz. Finally, we isolated responses in the different time windows described above and computed averaged responses


(4) $$X_{r}\,\left(s\right)=\frac{1}{N}\,\sum_{1}^{N}X_{d}\,\left(s+n\,T\right).$$

The smooth signal is shown in Figure 1C. We computed two mathematical quantities on the signal:

(1) The number *N_E_* of local extrema (minima and maxima) in the response attained at points *e_i_*.
(2) The total variation for the oscillation is measured by

(5) $$\left|\Delta \,V\right|=\sum_{i}\left|V\,\left(e_{i}\right)-V\,\left(e_{i+1}\right)\right|,$$

which is the sum of the absolute value of the difference between two consecutive extrema of the average evoked responses. This oscillation provides an information of the cumulative response amplitude; (ei) is the time point where the EEG signal is maximal or minimal.

#### Features identification associated to standard and deviant responses

**FIGURE 2:**
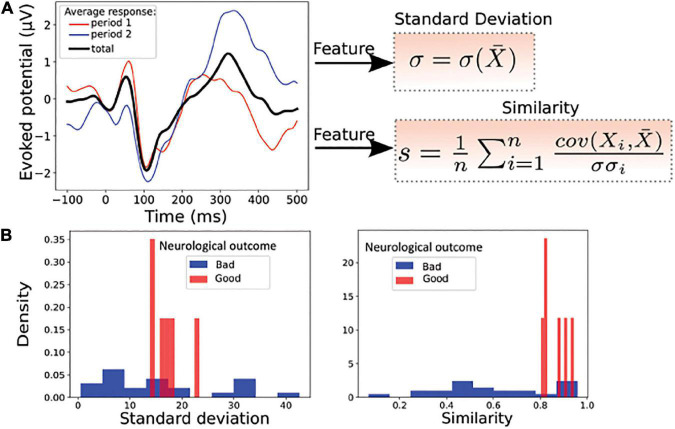
Statistical features associated to standard responses. **(A)** Left: average evoked responses computed over a time window of [0−10]*min* (period 1, red), [10−20]*min* (period 2, blue) and over the entire period ([0−20]*min*, black). Right: the standard deviation σ and the average correlation function *s*(similarity), between the response over the entire period ([0−20]*min*) and over one of the n periods ([0−20]*min* or [10−20]*min*), here *n* = 2. **(B)** Example of features distribution of dataset from the Cz electrode: standard deviation (Left) and similarity (Right) computed over the entire period; red (good neurological outcome) and blue (bad neurological outcome). These two parameters taken separately are insufficient to properly discriminate the two groups of patients.

For standard responses, we computed the standard deviation (formula 2) and the correlation function (formula 3) of the response computed between the response in the first and second time period (Figure 2A). To test the ability of these two parameters to separate the two categories of patients, we plotted the histogram of these two parameters for all patients in our data (Figure 2B), showing that each parameter individually could be potentially used for a classification.

**FIGURE 3:**
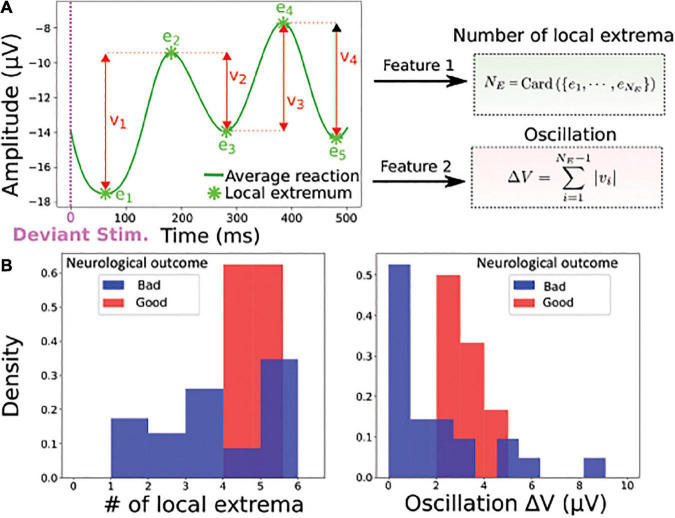
Statistical features associated to deviant responses. **(A)** Left: the average filtered evoked response (blue) to deviant stimuli computed over the entire time window contains *N_E_* local extrema *e_i_* (minimum or maximum), which is the first feature. The second feature is the oscillation |Δ*V*| = ∑_*i*_|*V*(*e*_*i*_)−*V*(*e*_*i* + 1_)|, which is the sum of the absolute value of the difference between two consecutive extrema of the average evoked response. **(B)** Example of feature distributions of dataset from all electrodes: local extrema (Left) and oscillation (Right) over the entire period; red (good neurological outcome) and blue (bad neurological outcome).

For the deviant responses, as the signal showed different characteristics, we decided to use novel features, the number of extremum *N_E_* present in the signal (Figure 3A) and the absolute value of the oscillation|Δ*V*|, which represents the sum of the differences between the extrema (formula 5). The result of this classification is shown by histograms of the two parameters computed over the whole population of patients (Figure 3B).

Although two different types of parameters were studied for standard and deviant responses, each of them taken individually was not sufficient to clearly separate the two categories of patients.

#### Classification using a two-dimensional map

Based on the parameters we extracted in the previous subsection, we generated two-dimensional maps: for the map associated to standard stimuli, each patient has the *P* = [σ_*X*_, *r*(*X*, *Y*)] coordinates, while for deviant stimuli, we used the *P* = (*N*_*E*_, |Δ*V*|) coordinates. In various plots, we normalized the coordinates in a population (*X*_1_, ..*X*_*n*_) by:


(6) $${\tilde{X}}_{i}=\frac{X_{i}-<X_{i}>}{\sqrt{Var\left(X_{1},..X_{n}\right)}},$$

Where *X_i_* is the average over the points *X_i_* and Var is the variance.

We mapped all points for all patients, where patients with bad *versus* good neurological outcome are shown in blue (vs red). Patients with good neurological outcome formed a cluster that will be the basis of the classification and prediction described below. The classification probability of a patient characterized by its coordinates was obtained by computing a score that measures the proximity to one of the two categories of patients.

To study the maps defined above as predictive tools, we used three independent statistical classifiers (SVM, Gaussian estimator, K-nearest neighbors). Because the present database did not contain many patients and to guarantee the robustness of our approach, we decided to use three classifiers (SVM, Gaussian mixture, and k-neighbors). As a small size database is also associated with overlearning, and to overcome this difficulty, we chose to use simple models for classification: Support Vector Machines (SVM) seems to be particularly suitable, as its classification is dependent only on a reduced number of patients. In fact, we wished to assign a good neurological outcome probability to any point that would be added on the map based on the ensemble of previous data points already classified. Using the assumption that statistics associated to patients (features) are independent from each other, we used a Bayesian classification.

##### SVM classification

To classify the data, we used the standard SVM algorithm (Cherkassky and Mulier, 1998), which determines the hyperplane that best separates the two classes. Briefly, the chosen hyperplane maximizes the distance between itself and the closest points of each class, while all points of a given class are located on one of the two sides (Valiant, 1984). If no such hyperplane is found, which is the case here, the dimension of the space where the data are embedded is increased, a procedure known as kernelling (Aizerman, 1964). In a higher dimensional space, the classes are well separated by a higher dimensional hyperplane. If the two classes are still not well separated, a penalty is inflicted for every misclassified data point (Cortes and Vapnik, 1995). Here, the kernel is the Radial Basis Function *K*(*x*, *x*′) = exp( − γ||*x* − *x*′||^2^), with γ=1 and a penalty coefficient *C* = 10. Note that we obtained similar confusion matrix for all pairs (γ,C)∈[0.5,2.5] × [3,30] for SVM.

We implemented the SVM using the Scikit Learn module (Pedregosa et al., 2011; Buitinck et al., 2013). Data analyses and classification codes were performed using Python.

##### Gaussian estimator

In case of a Gaussian estimator, we estimated the mean and the covariance matrix for the 2 categories of patients. The probability of each class is computed empirically using the maximum likelihood estimator (Supplementary methods). We recall that for an ensemble of n data 𝒮_*n*_ = (*x*_1_, ..*x*_*n*_) that are separated into two classes, *C_1_* and *C_2_*, the probability that a patient *X* belongs to one class, conditioned on the ensemble 𝒮*_n_*:


$$\;p\left(X\in C_{1}|X=x,{𝒮}_{n}\right)=$$

(7) $$\frac{1}{1+\frac{1-\Pi }{\Pi }\,\frac{{\left|\Sigma_{1}\right|}^{\frac{1}{2}}}{{\left|\Sigma_{1}\right|}^{\frac{1}{2}}}\,e\,x\,p\,\left(-\frac{1}{2}\,{\left(x-\mu_{1}\right)}^{T}\,\Sigma_{1}^{-1}\,\left(x-\mu_{1}\right)+\frac{1}{2}\,{\left(x-\mu_{2}\right)}^{T}\,\Sigma_{2}^{-1}\,\left(x-\mu_{2}\right)\right)}$$

where (μ_*i*_, Σ_*i*_)_*i* = 1,2_ are the mean and variance computed from each class *C_1_* and *C_2_* from 𝒮_*n*_. We used the fraction $\pi =\frac{n_{s}}{n_{s}+n_{d}}=$, *n_s_* for the number of patients with good neurological outcome at discharge and *n*_*d*_ for the other patients. Formula 7 is derived in the Supplementary methods.

##### K-nearest neighbors classifier and weighted K-nearest neighbors

To classify the standard stimuli, we used the K-nearest neighbors classifier. We computed the ratio for the probability of belonging to a class. For a given point *X*, the probability to belong to class *C_1_* (”good neurological outcome”) given the distribution of point *x* is computed empirically as the number of neighbors out of a total of K.


(8) $$p\left(X\in C_{1}|x\right)=\frac{k_{r}}{K}$$

where *k_r_* is the number of neighbors that belong to the class “good neurological outcome at discharge” among K closest points.

To classify deviant stimuli, we used a variant of the K-neighbors method by adding distance-relative weights to the points inside the dataset. The two classes labeled "*badneurologicaloutcome*" and "*goodneurologicaloutcome*" are defined as *C_1_* and *C*_*2*,_ respectively. The ensemble of points 𝒮_*n*_ in dimension 2 are given by the coordinates *x*_*n*_ = (*N*_*E*,*n*_, Δ*V*_*n*_), extracted in subsection “Analysis of responses to deviant stimuli.” To compute the classification probability, we defined K-nearest neighborhood 𝒩_*K*_(*x*) for the point *x* as the *K* shortest points from *x*, computed from the Euclidean distance (between two points *x*_*n*_, *x*_*m*_),


(9) $$d\,\left(x_{n},x_{m}\right)=\sqrt{{\left(N_{E,n}-N_{E,m}\right)}^{2}+{\left(\Delta \,V_{n}-\Delta \,V_{m}\right)}^{2}}$$

(10) $${𝒩}_{K}\left(x\right)=\left\{y_{1},..y_{K}\in {𝒮}_{n},d\left(x,y_{1}\right)\leq d\left(x,y_{1}\right)..\leq d\left(x,y_{K}\right)\right\}.$$

To obtain an accurate classifier, we used a different version of the K-neighbors classification, where the weight depends on the distance between the point to classify and the K-nearest neighbors (formula 11), defined by


(11) $$p\left(x\in C_{1}|x\right)=\frac{\sum_{i=1}^{N}\frac{1_{y_{i}\in C_{1}}}{d\,\left(y_{i},x\right)}}{\sum_{i=1}^{N}\frac{1}{d\,\left(y_{i},x\right)}}.$$

##### Cross-validation

We used a Leave-one-out cross-validation approach to validate the classification algorithm: we excluded a patient at a time and computed the probability of a good neurological outcome at discharge, based on the remaining elements in the data basis (Kohavi, 1995). In other words, we separated the patient database into a testing and a training group, with one patient out, 28 in the other group and ran this test 28 times so that each of the 29 patients was alternatively included in the 1 group patient. We then computed this probability using the three classifiers, SVM, Gaussian estimators and K-neighbors and compared the result to the true result. We followed the protocol: 1- a patient *P*_*i*_, *i* = 1..*N* is selected inside the data basis; 2- we trained the classification algorithm on the database of all patients {*P*_*k*_, *k* = 1..*N*} − *P*_*i*_. We evaluated the prediction of the model on the excluded patient, leading to a score *s_i_*. We recall that *s*_*i*_ = 1 if the prediction is correct, otherwise, *s_i_* = 0. We then replaced the patient *P_i_* inside the database and reiterated the procedure until each patient has been exactly excluded once. This allowed us to reclassify with a given probability for each patient outcome based on the new map determined by the other patients. The final score of the model is computed as


(12) $$s=\frac{1}{n}\,\sum_{1}^{n}s_{i}.$$

Finally, the confusion matrix defined as


(13) $$𝒞=\left(\begin{array}{cc} T_{p} & F_{N}\\ F_{p} & T_{N} \end{array}\right)$$

for the true positive *T_p_* (number of patients who have a good neurological outcome at discharge and are classified correctly), true negative *T_n_* (number of patients who have a bad neurological outcome and are classified correctly) and false positive *F_p_* (number of patients who have a good neurological outcome and are classified incorrectly) and false negative *F_n_* (number of patients who have a bad neurological outcome at discharge and are classified incorrectly). We calculated for each of the classifiers accuracy, sensitivity and specificity.

##### Combined probability for outcome decision

We proposed to use for the predictive decisional probability *p*_*dec*_ the minimum of the ones estimated for the standard (relation 8) and deviant (relation 11) classifications. For a patient of coordinate *x* in each map, survival probability is:


(14) $$p_{d\,e\,c}\left(x\in C_{1}|x\right)=\min\left(p_{d\,e\,v}\left(x\in C_{1}|x\right)\right),p_{n\,o\,n-d\,e\,v}\left(x\in C_{1}|x\right)).$$

##### Iteration and changing k-neighbors k

The approach developed here is iterative and any new additional case enriches the database and the classifications maps. For the K-neighbors approach, adding a point does not require any changes in the computation, although we expect that the number of neighbors that will enter progressively into the computation could diminish as the number of cases added in the map increases. For the Gaussian classification, the mean and the variance are recomputed following each new case.

## Results

### Overall patient characteristics

Data of twenty-nine consecutive patients were analyzed. Seven patients out of twenty-nine survived, but only 6 out of 7 were able to follow verbal command at hospital discharge. None of the patients was lost of follow-up. The last patient returned home but the degree of disability is unknown. At 3–6 months, GOS-E was scored at 3 for the patient who was unable to follow verbal command at discharge and died 27 months later without neurological improvement. GOS-E was scored at 4 for one patient, at 5 for one patient, at 6 for one patient and at 8 for the last two patients. Age, sex, medical history, characteristics of CA and electrophysiological features are presented in Table 1. At the time of recording, all patients were still hypothermic (<35°C). Sedation was present in 11 out of the 29 patients (38%) at the moment of the electrophysiological recordings. For the non-surviving patients, 18 out of 22 died after withdrawal of life-sustaining therapies.

**TABLE 1:** Comparison of clinical and electrophysiological characteristics between the two groups.

	Bad neurological outcome (*n* = 23 unless otherwise specified) (/29)	Good neurological outcome (*n* = 6) (/29)	*p*
Age (years), mean ± SD [min-max] Median [IQR 25–75]	60 ± 16 [24–87] 62 [54–68.5]	47.5 ± 16 [26–64] 52.5 [34.75–59]	0.07
Male, *n*	20	5	1
Shockable rhythm*, *n*	6	2	1
Etiology Cardiac Non-cardiac Undetermined	10 12 1	4 2 0	0.57
No-flow (minutes), mean ± SD Median [IQR 25–75]	8.3 ± 8.4 (/21) 7 [1–15]	2.7 ± 4.3 0 [0–4.5]	0.07
Low-flow (minutes), mean ± SD Median [IQR 25–75]	24.9 ± 16.4 20 [18.5–35]	16.4 ± 11.7 15.5 [10.75–21]	0.22
Time to ROSC (≤25 min), *n*	8 (/21)	4	0.20
GCS on admission /15), mean ± SD Median [IQR 25–75]	3.1 ± 0.4 3 [3–3]	3.5 ± 1.2 3 [3–3]	0.21
SAPS II score, mean ± SD Median [IQR 25–75]	73 ± 15 72 [63–85]	62.5 ± 18 54 [49–76]	0.15
Sedation, *n*	8	3	0.65
EEG Grade I: predominant alpha with some theta, *n*	0	1	
EEG Grade II: predominant theta with some alpha, *n*	0	0	
EEG Grade III: predominant theta, *n*	3	5	*0.0002*
EEG Grade IV: delta activity, *n* Generalized epileptiform periodic activity (GPEDs), *n*	7 6	0 0	
EEG Grade V electrocerebral silence, *n* Burst suppression patterns, *n*	3 4	0 0	
EEG reactivity, *n*	3	2	0.27
SSEP (N20 -), *n*	7 (/22)	1/5	0.64
AEP (MMN+), *n*	4	2	0.57
GOS-E (6 months) (*n*)	3 (1/23)	4–8 (5/6)	

*As the first documented rhythm; ROSC, return of spontaneous circulation; GCS, Glasgow Coma Scale; SAPS II score, simplified acute physiology score II; EEG, electroencephalography patterns according to the five major grades of severity scale for brain injury; SSEP, cortical somatosensory evoked potentials; AEP, auditory evoked potentials; MMN, mismatch negativity. No-flow data were missing in two patients and SSEP (N20 response) data in one patient (underlying Charcot Marie Tooth disease). GOS-E, Glasgow Outcome Scale-Extended.

All six patients with good neurological outcome presented an EEG pattern graded between I to III for all, whereas 20 out of the 23 of the patients with final bad neurological outcome or death presented an EEG pattern graded IV or V (*p* < 0.0002), including the patient who survived 27 months with bad neurological outcome. EEG reactivity (2/6 versus 3/23) and presence of MMN (2/6 versus 4/23) were more frequent in the group with good neurological outcome, whereas N20 was less frequently absent (1/5 versus 7/22), but none of these last markers were statistically different between the two groups. Only 2 patients presented congruent favorable prognostic factors with a present N20 at SSEP, a positive MMN and EEG pattern graded I to III (areactive EEG for both), among whom one patient did not survive. By contrast, four patients presented congruent bad prognosis factors with absent N20, absent MMN and an EEG pattern graded IV or V and all of them died. ERP obtained at Cz location were the most reproducible and the only ones used for visual analysis. Artifacts prevented the interpretation of SSEP in one patient of each group.

### Prognosis map constructed from bayesian statistical inference

**FIGURE 4:**
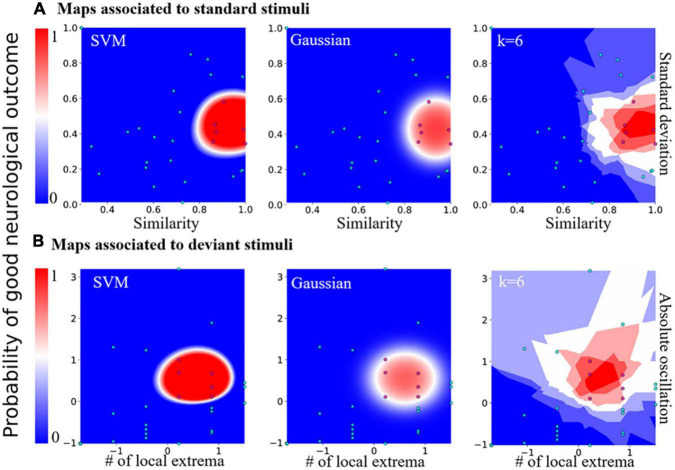
Predictive probability maps of good neurological outcome. **(A)** Probability maps computed from features of the standard stimuli responses. From left to right: maps computed from SVM, Gaussian, and the k-nearest neighbors classifier (*k* = 6, the worst case scenario). **(B)** Probability maps computed from features of the deviant stimuli features. From left to right: maps computed from SVM, Gaussian, and the k-nearest neighbors classifier with distance-related weights, *k* = 6 for example.

Since each parameter taken individually for standard (standard deviation and similarity) and deviant (number of extremum *N_E_* and oscillation|Δ*V*|) responses were not sufficient to obtain a clear separation between the two patient categories, we decided to combine them into a two-dimensional map (Figure 4). Interestingly, we found that this map allowed a clear separation that we quantified using various *a priori* classifiers: SVM, Gaussian, and the K-neighbor classifiers (Hastie et al., 2001; Table 2). When mapping all the features first taken individually for standard and deviant responses, we found a cluster formed of patients with a “good” neurological outcome, bounded in red, well separated from the area in which were found the other patients (non-surviving or “bad” neurological outcome). This partition between two distinct areas was present in all classifiers: SVM, Gaussian, and k-neighbors, confirming that this partition was robust independently of the choice of the classification methods (Supplementary Figure 1 for other choices of k for the k-neighbor algorithm). Moreover, we found a similar partition into two categories of patients when classifying the standard or the deviant responses, which strengthens the robustness of our study (Figure 4). The present classification maps for both standard and deviant responses studied separately showed that the neurological outcome of post-anoxic comas can be predicted (Table 2). Combining the probability computed in each map, we proposed a decision probability with a high specificity, which does not misclassify patients with good neurological outcome in the category of patients with bad neurological outcome.

**TABLE 2:** Accuracy, sensitivity, and specificity obtained by cross-validation for responses to standard, deviant stimuli; k is the number of neighbors used in the classification algorithm.

	*k* = 3	*k* = 4	*k* = 6	*k* = 8	SVM	Gaussian
**Accuracy**						
Standard responses	0.96	0.96	0.89	0.85	1.0	0.92
Deviant responses	0.89	0.89	0.89	0.89	1.0	0.89
**Sensitivity**						
Standard responses	0.83	0.83	0.83	0.66	1.0	0.66
Deviant responses	0.83	0.83	0.83	0.83	1.0	0.5
**Specificity**						
Standard responses	1.0	1.0	0.9	0.9	1.0	1.0
Deviant responses	0.91	0.91	0.91	0.91	1.0	1.0

### Classification efficiency of the two-dimensional maps

To test the predictive strength of the standard and deviant responses classification, we computed the confusion matrix (formula 13) as described in the methods. The confusion matrix computed for the Gaussian estimator showed a 89 % accuracy, and a 100% validation accuracy for the SVM classifier. The confusion matrix computed for the k-neighbors classifier showed that it was less performant than the SVM classifier. The sensitivity remained high and could be improved with the increasing number of classified patients (*k* = 4; similar results were obtained for other values of k). The distance-dependent weight showed this estimator introduces type I error, with an accuracy of 0.90, a sensitivity of 0.83 and a specificity of 0.91.

Finally, we also computed the confusion matrix obtained from a visual analysis of patient MMN, performed by a medical professional, and we obtained an accuracy of 0.72, a sensitivity of 0.33 and a specificity of 0.82. If MMN remained an interesting indicator, it showed a very weak sensitivity in these patients (Tables 2, 3).

**TABLE 3:** Classifications scores.

	Accuracy	Sensitivity	Specificity
SVM	1	1.0	1
Gaussian	0.89	0.5	1
k-neighbors	0.9	0.83	0.91
MMN	0.72	0.33	0.82

## Discussion

Our exploratory study was designed to identify mathematical parameters extracted from the AEP recording that could be more powerful than visual inspection of MMN in the routine ICU setting and used to predict neurological prognosis in these patients. The originality of the present strategy was to consider independently deviant from standard responses, not only in the time window of the mismatch negativity (that results from the difference between the two responses), but using the total amount of information that is generated during the procedure. We found that our new classification method, combining standard deviation and similarity (correlation) for standard auditory stimuli, and number of extrema and oscillations for deviant auditory stimuli, allowed clustering patients in two-dimensions, in one of the two categories of good or bad neurological prognosis. Importantly, we did not select these parameters *a priori* to obtain a best separation of patients as explained in the method’s section.

To evaluate the robustness of our method, we used three classifiers, showing similar maps classification results. Finally, using leave-one-out cross-validation, we computed a score for each classifier, demonstrating that any of the three classification methods was more robust than simply analyzing the MMN in a binary response, using logistic regression or single-trial topographic analysis (De Lucia and Tzovara, 2015). We showed that good neurological prognosis probability maps allow us to predict the neurological outcome of post-anoxic comatose patients with a very good accuracy of 0.90, sensitivity of 0.83 and specificity of 0.91 when considering the least efficient classifier (Tables 2, 3).

We have used the standard deviation and the similarity index to analyze the standard responses, while we used the number of extrema and oscillations for the deviant in order to have two independent set of parameters and increase the robustness of the results, and preventing a potential bias by choosing a single set of parameters. We could have decided to use these two latest parameters in this study for all cases or use all four parameters that could have led to a more robust result, but also to a four-dimensional classification, that we wanted to avoid in order to obtain an easy-to-use tool. Moreover, the standard deviation and the similarity index would not really be appropriate to study the deviant sounds.

We can consider three other developments that could be built on this present investigation. The first one is to evaluate if repeating this procedure with this algorithm several days apart can present a potential additive value, as explained in Tzovara et al. (2013) who showed the additional prognostic value of repeating MMN. The second one is to test whether this procedure could be generalized to other auditory oddball paradigms. At last, it would be interesting to evaluate whether such a method could be applied to classical electroencephalography with more sparse auditory and nociceptive stimuli than the one developed here using auditory evoked potentials with frequent and regular auditory stimuli. Indeed, electroencephalography is a neurophysiological tool which is more widespread than auditory evoked potentials. Characterizing electrophysiological features to predict the outcome of post-anoxic coma remains a genuine challenge. There is currently no satisfactory, efficient and simple tool to predict comatose patient outcome accurately, especially at the acute phase, when patients are sedated and/or hypothermic. Standard electroencephalography is the most common method used to predict prognosis in those patients. If highly malignant pattern (suppressed background discharges without discharges or with continuous periodic discharges, or burst suppression background with or without discharges) is highly specific of poor outcome, as shown in our study, it has a sensitivity of only 50% (Westhall et al., 2016). The absence of cortical N20 response at SSEP after stimulation of median nerves has an almost 100% specificity for non-awakening prediction (Sandroni et al., 2014). By contrast, the predictive value of the visual analysis of MMN for post-CA comatose patients, limited to a binary response (presence/absence of a detectable peak of the MMN between the standard and deviant responses) is poorly sensitive, as shown in our study, even when choosing parameters that better discriminate standard and deviant sounds (Azabou et al., 2018). To overcome, the poor sensitivity of MMN at visual analysis, several statistical methods have been developed. Some are based on sample-by-sample paired *t*-test in the specific time window where MMN is ussually visualized. Others are based on wavelet transform, multivariate, cross-correlation and probabilistic methods (Fischer et al., 1999; Naccache et al., 2005, 2015; Daltrozzo et al., 2007; De Lucia and Tzovara, 2015, 2016; Gabriel et al., 2016; Juan et al., 2016). Tzovara et al. (2013) choose an alternative strategy: they showed that the progression of MMN auditory discrimination (and not one single analysis) over the first 2 days of coma was of good prognosis, suggesting that collecting repetitive data within days, or at an earlier phase, could reveal changes that could have a higher predictive value. Overall, this explains why a multimodal prognostication approach is still recommended in these patients, including clinical examination, serum biomarkers and brain imaging in addition to electrophysiological recordings (Sandroni et al., 2014; Nolan et al., 2021).

In that small series, none of the classical electrophysiological tools were sensitive or specific enough to give a reliable neurological prognosis. Only 2 patients presented congruent favorable prognostic factors with a present N20 at SSEP, a positive MMN and EEG pattern graded I to III (benign pattern according to the ACNS EEG terminology) (Westhall et al., 2015) and areactive for both, among whom one patient did not survive. By contrast, four patients presented congruent bad prognosis factors with absent N20, absent MMN and an EEG pattern graded IV or V (highly malignant pattern according to the ACNS EEG terminology) and all of them died, suggesting that congruent pejorative factors are strongest indicators of prognosis than congruent good prognosis factors, in accordance with literature. It is to note that one third of the patients with bad outcome and 50% of the patients with good outcome were under sedation at the time of recording, which is known to impede electrophysiology interpretation. Our study was not designed to compare our tool with classical electrophysiological examinations but sensitivity and specificity were higher in that small cohort that needs to be validated in a larger cohort. The total amount of data we collected for all epochs during the 20 min of auditory stimulations and not only during the time window used for MMN might explain our more sensitive results.

Our present study has several limitations. First, as a retrospective study, neurological prognosis was evaluated on the ability of the patient to follow verbal command at discharge, which remains a subjective assessment that may have led to patient’s misclassification. However, in the 7 surviving patients, GOS-E was available for 6 of them at 3–6 months post-discharge and was found at 3 in the patient who was initially unable to follow verbal command and from 4 to 8 for the others, indicating that no patient was initially misclassified. Second, this cohort may not be representative of all post CA patients since electrophysiological assessment was performed relatively late, up to 6 days after admission, in patients still comatose at the time of the evaluation, and the relatively small sample size prevents generalization of our results that need to be replicated in a larger cohort. Third, our cohort between patients with good and bad prognosis was unbalanced, that we tried to offset using a leave-one out cross validation. Fourth, our new approach did not consider the order of the different sounds. For instance, a standard sound that would start a new sequence just after a deviant sound or ending a series of standard sounds just before a deviant sound, may not be processed the same. This point could deserve a specific attention in future studies, but as we averaged all our data, this probably does not bias our results.

To conclude, we developed a new promising classification method that could be self-sufficient, easily used by intensivists (only one electrode, with minimal cost and easy training), without the help of the neurophysiologists and in sedated and/or hypothermic patients, since these conditions represent actual limitations to electrophysiological data acquisition in the ICU. Moreover, electrophysiological recordings may be particularly difficult to acquire at the acute phase where patients combine aggressive care (extracorporeal membrane oxygenation (ECMO), haemodialysis, mechanical ventilation), and invasive methods of monitoring, generating artifacts. Finally, potential amplitudes are smaller under sedation and more difficult to extract from the background (Yppärilä et al., 2004). Our preliminary results suggest that all these issues could be addressed by this new method. The produced maps can be refined and upgraded by adding new cases and thus increase the performance of the probabilistic classifier. In the future, and according to the local human and logistical resources, the software could be implemented with other electrophysiological and clinical variables to provide an optimal estimated probability of the patient outcome, independently from neurophysiologists. Developing such algorithms, ready-to-use by the intensivits, would enable more aggressive management in patients with predicted good neurological outcome. Whether this approach could be secondarily applied to other predictive situations and generalized to other comas remains to be validated.

## Data availability statement

The raw data supporting the conclusions of this article will be made available by the authors, without undue reservation.

## Ethics statement

The studies involving human participants were reviewed and approved by the Ethics Committee (Comité de Protection des Personnes, CPP Paris IV #2012/22) (PHRC CAPACITY AOR10109). Informed consent was provided by next-of-kin for all participants as they were in a coma most of the time until their death, it was followed whenever possible, by informed consent from the patient.

## Author contributions

AF, AD, and DH created the algorithm. SL, ND, and BM collected the clinical data. AR, EM, SM, PL, and NK performed the electrophysiological examinations. DH and NK wrote the manuscript. All authors read and approved the final manuscript.
