# Supplementary figures and images for: Predicting neurological outcome after cardiac arrest by combining computational parameters extracted from standard and deviant responses from auditory evoked potentials
Source: Front Neurosci. 2023 Feb 15;17:988394. doi: 10.3389/fnins.2023.988394 (PMC9975713; doi:10.3389/fnins.2023.988394)

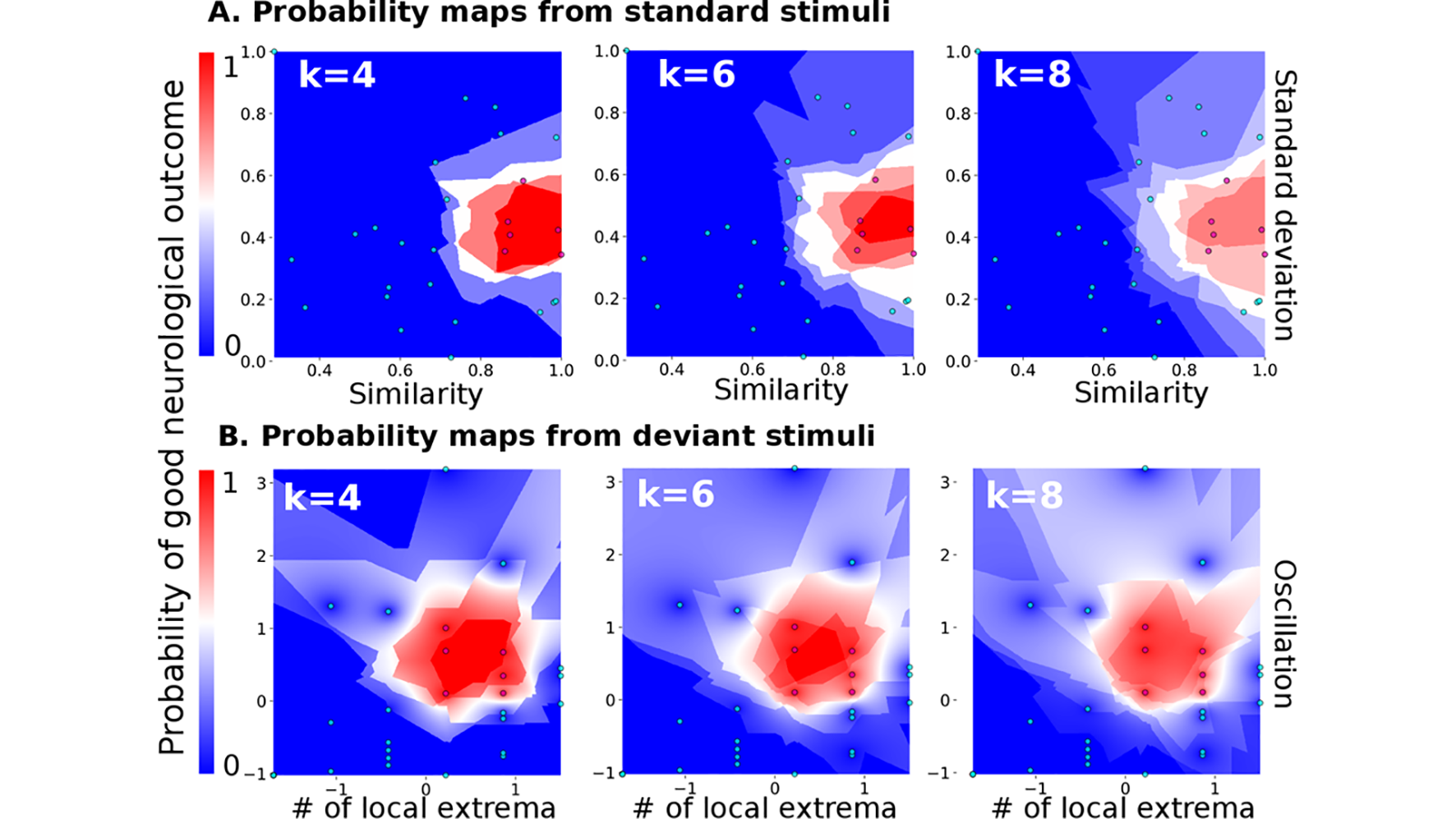

Supplement: Supplementary file 2 [file Image_1.tif]
